# Supplementary figures and images for: Endoscopic application of mussel-inspired phenolic chitosan as a hemostatic agent for gastrointestinal bleeding: A preclinical study in a heparinized pig model
Source: PLoS One. 2021 May 14;16(5):e0251145. doi: 10.1371/journal.pone.0251145 (PMC8121352; doi:10.1371/journal.pone.0251145)

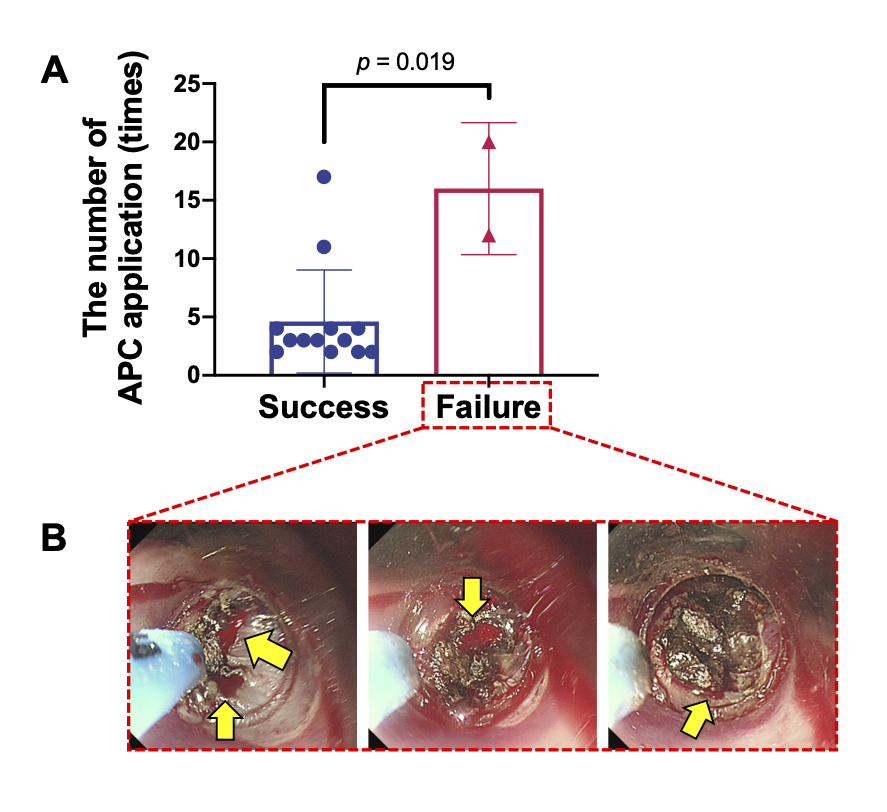

Supplement: S1 Fig — (A) The number of APC applications (times). (B) Photographic images of the failure cases at 2 min after APC application (yellow arrows indicate bleeding). (TIF) [file pone.0251145.s003.tif]
