# Supplementary material for: Endoscopic application of mussel-inspired phenolic chitosan as a hemostatic agent for gastrointestinal bleeding: A preclinical study in a heparinized pig model
Source: PLoS One. 2021 May 14;16(5):e0251145. doi: 10.1371/journal.pone.0251145 (PMC8121352; doi:10.1371/journal.pone.0251145)
Supplement: Attachment — Submitted filename: Response to Reviewers.docx [file pone.0251145.s004.docx]

**PONE-D-20-38401**

**Endoscopic application of mussel-inspired phenolic chitosan as a hemostatic agent for gastrointestinal bleeding: a preclinical study in a heparinized pig model**

PLOS ONE

Dear Dr. Lee,

Thank you for submitting your manuscript to PLOS ONE. After careful consideration, we feel that it has merit but does not fully meet PLOS ONE’s publication criteria as it currently stands. Therefore, we invite you to submit a revised version of the manuscript that addresses the points raised during the review process.

Please submit your revised manuscript by Apr 17 2021 11:59PM. If you will need more time than this to complete your revisions, please reply to this message or contact the journal office at *plosone@plos.org*. When you're ready to submit your revision, log on to <https://www.editorialmanager.com/pone/> and select the 'Submissions Needing Revision' folder to locate your manuscript file.

Please include the following items when submitting your revised manuscript:

- A rebuttal letter that responds to each point raised by the academic editor and reviewer(s). You should upload this letter as a separate file labeled 'Response to Reviewers'.
- A marked-up copy of your manuscript that highlights changes made to the original version. You should upload this as a separate file labeled 'Revised Manuscript with Track Changes'.
- An unmarked version of your revised paper without tracked changes. You should upload this as a separate file labeled 'Manuscript'.

If you would like to make changes to your financial disclosure, please include your updated statement in your cover letter. Guidelines for resubmitting your figure files are available below the reviewer comments at the end of this letter.

If applicable, we recommend that you deposit your laboratory protocols in protocols.io to enhance the reproducibility of your results. Protocols.io assigns your protocol its own identifier (DOI) so that it can be cited independently in the future. For instructions see: <http://journals.plos.org/plosone/s/submission-guidelines#loc-laboratory-protocols>

We look forward to receiving your revised manuscript.

Kind regards,

Panayiotis Maghsoudlou
Academic Editor
PLOS ONE

***Journal Requirements:***

*Please review your reference list to ensure that it is complete and correct. If you have cited papers that have been retracted, please include the rationale for doing so in the manuscript text, or remove these references and replace them with relevant current references. Any changes to the reference list should be mentioned in the rebuttal letter that accompanies your revised manuscript. If you need to cite a retracted article, indicate the article’s retracted status in the References list and also include a citation and full reference for the retraction notice.*

*When submitting your revision, we need you to address these additional requirements.*

1. *Please ensure that your manuscript meets PLOS ONE's style requirements, including those for file naming. The PLOS ONE style templates can be found at*

[*https://journals.plos.org/plosone/s/file?id=wjVg/PLOSOne_formatting_sample_main_body.pdf*](https://journals.plos.org/plosone/s/file?id=wjVg/PLOSOne_formatting_sample_main_body.pdf)

*and*

[*https://journals.plos.org/plosone/s/file?id=ba62/PLOSOne_formatting_sample_title_authors_affiliations.pdf*](https://journals.plos.org/plosone/s/file?id=ba62/PLOSOne_formatting_sample_title_authors_affiliations.pdf)

**Answer:**

The authors checked the manuscript meets PLOS ONE’s requirements.

2. *We note that you have provided ethics information within your manuscript. However, we ask that you also provide it in the metadata that you are asked to fill out when you resubmit. This is the section where you are prompted to fill in the title, abstract data availability statement, etc. One of these sections is labelled "Ethics Statement". You have currently written that the information has been included in the manuscript. Please change this to:*

*'This study was approved by the Institutional Animal Care and Use Committee of KNOTUS Co., Ltd. (KNOTUS IACUC, approval no.16-KE-200) and conducted in compliance with Good Laboratory Practice (GLP) regulations (21 CFR Part 58, US FDA). The animals were sedated by intramuscular injection with a mixture of tiletamine/zolazepam (Zoletil® 95, Virbac, 5 Carros, France) and xylazine (Rompun® 96, Bayer, Leverkusen, Germany). General anesthesia was maintained by mechanical ventilation with 1% to 2% isoflurane (Forane® 97, Baxter, Deerfield, IL, USA) in oxygen. If humane endpoints were reached, such as significant weight loss exceeding 20% of initial baseline body weight, lethargy, pyrexia, hypothermia, or behavior that signals severe pain, the animals were sacrificed and excluded from this study. The animals were humanely euthanized by intravenous (IV) injection of suxamethonium chloride (Succicholine Inj® 50 mg/mL, Ilsung Pharmaceuticals, Seoul, Republic of Korea) at a dose of 0.1 mL/kg under deep anesthesia.'*

**Answer:**

As following the editor’s suggestion, we added the information in the metadata when we resubmit.

3. *Thank you for stating the following in the Acknowledgments Section of your manuscript:*

*"This study was supported in part by a grant from the National R&D Program for Cancer Control, the Ministry for Health and Welfare, Republic of Korea (HA16C0016 (1631060), H.L.)."*

*We note that you have provided funding information that is not currently declared in your Funding Statement. However, funding information should not appear in the Acknowledgments section or other areas of your manuscript. We will only publish funding information present in the Funding Statement section of the online submission form.*

*Please remove any funding-related text from the manuscript and let us know how you would like to update your Funding Statement. Currently, your Funding Statement reads as follows:*

*"The funders had no role in study design, data collection and analysis, decision to publish, or preparation of the manuscript."*

*Please include your amended statements within your cover letter; we will change the online submission form on your behalf.*

**Answer:**

As following the editor’s suggestion, we removed the funding source in the acknowledgments and updated the Funding Statement, as shown below.

[Page 18, Lines 411-414]

[Before]

**Acknowledgments**

This study was supported in part by a grant from the National R&D Program for Cancer Control, the Ministry for Health and Welfare, Republic of Korea (HA16C0016 (1631060), H.L.). We would like to thank to Ji-Hee Kim for helping with the pig experiment and filming the experiment and the veterinary team of Knotus Co., Ltd. for their help all along the animal care and experiment supports.

[After]

**Acknowledgments**

We would like to thank Ji-Hee Kim for helping with the pig experiment and filming the experiment and the veterinary team of Knotus Co., Ltd. for their help all along with the animal care and experiment supports.

[Updated in the cover letter]

**Funding Statement**

This study was supported by a grant from the National R&D Program for Cancer Control of the Ministry for Health and Welfare, Republic of Korea (Grant No. HA16C0016 (1631060), H.L.). The funder had no role in study design, data collection and analysis, decision to publish, or preparation of the manuscript. InnoTherapy Inc. only provided financial support in the form of authors salaries (KK, MYK, MSL, and HL) and research materials (CHI-C hemostatic agent), but did not have any additional role in the study design, data collection and analysis, decision to publish, or preparation of the manuscript. The specific roles of these authors are articulated in the ‘authors contributions’ section.

4. *Thank you for stating the following in the Competing Interests section:*

*"NO authors have competing interests."*

*We note that one or more of the authors are employed by a commercial company: InnoTherapy Inc..*

4.1. *Please provide an amended Funding Statement declaring this commercial affiliation, as well as a statement regarding the Role of Funders in your study. If the funding organization did not play a role in the study design, data collection and analysis, decision to publish, or preparation of the manuscript and only provided financial support in the form of authors' salaries and/or research materials, please review your statements relating to the author contributions, and ensure you have specifically and accurately indicated the role(s) that these authors had in your study. You can update author roles in the Author Contributions section of the online submission form.*

*Please also include the following statement within your amended Funding Statement.*

*“The funder provided support in the form of salaries for authors [insert relevant initials], but did not have any additional role in the study design, data collection and analysis, decision to publish, or preparation of the manuscript. The specific roles of these authors are articulated in the ‘author contributions’ section.”*

*If your commercial affiliation did play a role in your study, please state and explain this role within your updated Funding Statement.*

**Answer:**

As following the editor’s suggestion, we rewrote the Funding statement as follows:

[Updated in the cover letter]

**Funding statement**

This study was supported by a grant from the National R&D Program for Cancer Control of the Ministry for Health and Welfare, Republic of Korea (Grant No. HA16C0016 (1631060), H.L.). The funder had no role in study design, data collection and analysis, decision to publish, or preparation of the manuscript. InnoTherapy Inc. only provided financial support in the form of authors salaries (KK, MYK, MSL, and HL) and research materials (CHI-C hemostatic agent), but did not have any additional role in the study design, data collection and analysis, decision to publish, or preparation of the manuscript. The specific roles of these authors are articulated in the ‘authors contributions’ section.

4.2. *Please also provide an updated Competing Interests Statement declaring this commercial affiliation along with any other relevant declarations relating to employment, consultancy, patents, products in development, or marketed products, etc.*

*Within your Competing Interests Statement, please confirm that this commercial affiliation does not alter your adherence to all PLOS ONE policies on sharing data and materials by including the following statement: "This does not alter our adherence to PLOS ONE policies on sharing data and materials.” (as detailed online in our guide for authors* [*http://journals.plos.org/plosone/s/competing-interests*](http://journals.plos.org/plosone/s/competing-interests)*). If this adherence statement is not accurate and there are restrictions on sharing of data and/or materials, please state these. Please note that we cannot proceed with consideration of your article until this information has been declared.*

*Please include both an updated Funding Statement and Competing Interests Statement in your cover letter. We will change the online submission form on your behalf.*

*Please know it is PLOS ONE policy for corresponding authors to declare, on behalf of all authors, all potential competing interests for the purposes of transparency. PLOS defines a competing interest as anything that interferes with, or could reasonably be perceived as interfering with, the full and objective presentation, peer review, editorial decision-making, or publication of research or non-research articles submitted to one of the journals. Competing interests can be financial or non-financial, professional, or personal. Competing interests can arise in relationship to an organization or another person. Please follow this link to our website for more details on competing interests:* [*http://journals.plos.org/plosone/s/competing-interests*](http://journals.plos.org/plosone/s/competing-interests)

**Answer:**

We apologize for our mistake in the Competing Interests section. We would like to revise the Competing interest as follows.

[Before]

**Competing Interests Statement**

NO authors have competing interests.

[After]

**Competing Interests Statement**

The authors have read the journal’s policy and have the following competing interests: Keumyeon Kim and Mi-Young Koh are employees of InnoTherapy Inc. Moon Sue Lee is a founder and owner of InnoTherapy Inc. Haeshin Lee is a co-founder of Innotherapy Inc. and serves as a Chief Technology Officer of InnoTherapy Inc. This does not alter our adherence to PLOS ONE policies on the sharing of data and materials.

5. *PLOS requires an ORCID iD for the corresponding author in Editorial Manager on papers submitted after December 6th, 2016. Please ensure that you have an ORCID iD and that it is validated in Editorial Manager. To do this, go to ‘Update my Information’ (in the upper left-hand corner of the main menu), and click on the Fetch/Validate link next to the ORCID field. This will take you to the ORCID site and allow you to create a new iD or authenticate a pre-existing iD in Editorial Manager. Please see the following video for instructions on linking an ORCID iD to your Editorial Manager account:* [*https://www.youtube.com/watch?v=_xcclfuvtxQ*](https://www.youtube.com/watch?v=_xcclfuvtxQ)

**Answer:**

As following the editor’s suggestion, the ORCID iDs of the corresponding authors (HL and JYC) have been verified in the Editorial Manager.

**Reviewers' comments:**

Reviewer's Responses to Questions

**Comments to the Author**

**Reviewer #1:** *This paper is generally well-written and provides a thorough assessment of the new catechol-chitosan wound patch technology. The paper thoroughly addresses both bleeding as well as wound healing, and the methods for the most part are well done. Thus, I have only a few critiques and requests for clarification:*

1. *It is unclear why an aPTT was run rather than a PT. While both reflect the effect of heparin on slowing coagulation, PT better reflects the ability of clot to form in tissue, while aPTT reflects the speed of clotting at artificial surfaces. Thus, the aPTT is a good measure for what might occur close to the gauze or the CHI-C patch, the PT is a better reflection of the tendency of the injury to bleed.*

**Answer**:

First of all, thank you for the reviewer’s comments. However, the authors disagree with the reviewer’s suggestion to use PT rather than aPTT to reflect the effect of heparin. In this study, we used unfractionated heparin (UFH, GC Pharma, Republic of Korea). In clinical practice, the aPTT is extensively used to monitor the treatment effect of unfractionated heparin, whereas the PT is used to monitor the anticoagulant effects of warfarin and other vitamin K antagonists (VKA) [Reference: Levy JH, Szlam F, Wolberg AS, Winkler A. Clinical use of the activated partial thromboplastin time and prothrombin time for screening: a review of the literature and current guidelines for testing. *Clin Lab Med*. 2014;34(3):453-477. doi:10.1016/j.cll.2014.06.005]. This is the main reason we used the aPTT, not the PT.

There is a report that PT was not significantly influenced by heparin dose in patients with thrombosis [Reference: Chen CC, You JY, Ho CH. The aPTT assay as a monitor of heparin anticoagulation efficacy in clinical settings. *Adv Ther.* 2003;20(5):231-236].

There are also monitoring tests that specifically measure UFH levels, a protamine titration test and an anti-factor Xa activity test. These methods have advantages such as that the measured value is uniform and standardized, but it is not commonly used in clinical practice due to difficult implementation and not easy automation.

In our study, the PT was also tested, and the results of the aPTT and the PT are shown in Table as follows. As a result, the change in aPTT values after heparin injection is greater than that of PT.

| **Table. PT and aPTT values** | | | |
| --- | --- | --- | --- |
| **Tests** | **Units** | **Baseline** | **5 min after the heparin inj.** |
| PT | sec | 15.7 ± 3.4 | 23.9 ± 6.7 |
| aPTT | sec | 18.3 ± 0.6 | 116.7 ± 18.6 |
| N | | 15 | 15 |

2. *The heparin was given as a bolus, rather than a drip, and thus the aPTT is falling over time, as acknowledged in the paper. Because of this, it is important to denote in what order the interventions were performed (APC, gauze, and CHI-C). Was it randomized or in a specific order? It should be clear if certain interventions faced a more challenging bleed. If so, that could potentially damage the findings for the hemostasis portion of the study.*

**Answer**:

The reviewer’s opinion is an important subject. Indeed, the test materials were applied in a random order. As for the question regarding ‘~ if certain interventions faced a more challenging bleed.’, the test materials were applied only when the initial bleeding met the Forrest Ib classification. The judgement was performed by a MD specialist by observing initial bleeding after the EMR procedure.

This information was already described in the Materials and Method in the original manuscript, as shown below (highlighted in yellow).

[Page 6, Lines 137-139]

Each pig received at least four EMR-induced injuries, and four test groups (no-treatment, gauze, APC, and CHI-C) were applied in random order.

[Page 9, Lines 192-193]

If the bleeding was more severe or milder than Forrest Ib, it was excluded from this study.

(The authors’ response continues…)

As for the question about ‘heparin was given as a bolus, rather than a drip, ~’, the investigators considered the half-life of heparin about 90 minutes. Thus, all procedures were completed within this time scale. This was described in the original manuscript as well.

[Page 8, Lines 185-186]

Since heparin has a half-life of about 90 minutes, all procedures to evaluate the hemostatic effect were completed within 90 minutes after heparin injection [28].

3. *Some of healing data is examined serially in one subject over multiple days. Thus, you should have a statistical method with repeated measures that incorporates a subject variable. From what I can tell, you haven’t done this. For continuous data, such as the % healing measurement, I would used a mixed model because it is better when you have some lost data (which I think you have from one pig). I am not as familiar with non-parametric statistics with repeated measures, so I recommend consulting a statistician to find the ideal repeated measures method for the ranked healing scores.*

**Answer**:

As the reviewer suggested, the authors had a consulting section with a statistician specialized in animal and clinical samples. The statistician confirmed that the shown healing scores were from the repeated measurement with missing (lost) data. The statistician performed repeated measures one-way ANOVA with us using a mixed-effects model (with the missing data) method to demonstrate whether the healing scores shown in day 1, 5, and 10 were statistically meaningful. *P* < 0.0001 value was obtained from the analysis indicating that the healing scores are statistically meaningful.

Thus, the authors added the following sentences considering reviewer’s comments.

[Page 11, Lines 229-238]

[Before]

**Statistical analysis**

Continuous variables are expressed as mean ± standard deviation (SD). The changes in aPTT values after heparin injection were compared using the paired *t*-test. Other continuous variables were analyzed using the Mann-Whitney *U* test because of the small sample sizes (non-parametric data). Categorical variables are reported using the proportion (%) and were analyzed using Fisher’s exact test. A *p*-value of less than 0.05 was considered statistically significant. Statistical analyses were performed using Prism version 8.2.0 (GraphPad Software Inc., San Diego, California, USA).

[After]

**Statistical analysis**

Continuous variables are expressed as mean ± standard deviation (SD). The changes in aPTT values after heparin injection were compared using the paired *t*-test. Repeated measures one-way ANOVA using a mixed-effects model (with the missing data) were performed to compare the healing score of CHI-C over the follow-up endoscopy. Other continuous variables were analyzed using the Mann-Whitney *U* test because of the small sample sizes (non-parametric data). Categorical variables are reported using the proportion (%) and were analyzed using the Fisher’s exact test. A *p*-value of less than 0.05 was considered statistically significant. Statistical analyses were performed using Prism version 8.2.0 (GraphPad Software Inc., San Diego, California, USA).

4. *The discussion is a bit unfocused, particularly in its discussion of the healing results. These are scattered over several different paragraphs. Bringing them together and more thoroughly discussing these results in one place would improve the discussion. Thus, I recommend:*

*P1. In the first paragraph, summarize your findings that CHI-C and APC are the best at stopping bleeding, but that APC causes more tissue injury. Exhaustively discuss the differences in tissue injury here, rather than spread over the other paragraphs.*

*P2. Then talk about the other advantages you see with CHI-C - accessibility*

*P3. Then limitations.*

**Answer**:

As following the reviewer’s suggestion, we revised the discussion section, as shown below. We followed the reviewer’s direction of the suggested logic flow.

**The 1^st^ and 2^nd^ paragraph**

[Page 16, Lines 361-382]

[Before]

**Discussion**

GI bleeding shows the risk of re-bleeding in up to 25% cases [5]. Complete prevention of re-bleeding remains challenging, and re-bleeding is associated with a high mortality rate. As shown in Fig 3B, Fig 4A and B, instant and rapid hemostasis by APC treatment has been proven to be effective, but the occurrence of re-bleeding was unavoidable as demonstrated in the heparinized porcine model. Thus, hemostasis via APC cannot solve the problem. Fundamentally, APC-mediated hemostasis causes tissue damage given the principle behind this treatment. Electrical energy is directly transferred to target tissues and produces significant heat in the form of electrical current. Thus, heat-induced denaturation of proteins generates a significant amount of fibrous tissue (red arrow, Fig 5A), which is not suitable to prevent re-bleeding (Fig 4A and B).

Recently, we reported tissue healing processes using a CHI-C conjugate for application as intestinal anastomotic sealants [24]. In that study, CHI-C was applied to the partial anastomotic site of the intestine to prevent leakage. CHI-C exhibited strong tissue adhesion as well as rapid tissue healing properties. Tissue adhesion shown by CHI-C is closely associated with its robust binding with proteins [23], and bound proteins enhance the functions of inflammatory cells such as fibroblasts. Subsequently, the series of tissue/material reactions promote granulations and re-organization, resulting in accelerated wound healing. Thus, CHI-C is beneficial for rapid healing, which might be responsible for its effective prevention of re-bleeding compared to the prevention efficacy of APC-involved healing. In addition to the tissue healing-related re-bleeding issues, the CHI-C group showed 93.3% hemostasis within 2 min. Thus, CHI-C is not inferior to APC for instant hemostasis as well.

[After]

**Discussion**

GI bleeding shows the risk of re-bleeding in up to 20% cases [5]. Complete prevention of re-bleeding remains challenging, and re-bleeding is associated with a high mortality rate. Our results showed that CHI-C and APC groups showed similar 2 min hemostasis: 93.3% (14/15) in the CHI-C group and 86.7% (13/15) in the APC group. However, APC-mediated hemostasis causes tissue damage given the principle behind this treatment. Electrical energy is directly transferred to target tissues and produces significant heat in the form of electrical current. Thus, heat-induced denaturation of proteins generates a significant amount of fibrous tissue (red arrow, Fig 5A), which is somewhat related to occurrence of the re-bleeding results shown in Fig 4A and B.

In contrast to the significant level of tissue damage caused by APC treatment, the use of CHI-C showed natural healing with no damage of tissues. Recently, we reported tissue healing processes using a CHI-C conjugate for application as intestinal anastomotic sealants [23]. In that study, CHI-C was applied to the partial anastomotic site of the intestine to prevent leakage. CHI-C exhibited strong tissue adhesion as well as rapid tissue healing properties. Tissue adhesion shown by CHI-C is closely associated with its robust binding with proteins [23,24,26], and the bound proteins enhance the functions of inflammatory cells such as fibroblasts. Also, hepatectomy clinical trial using CHI-C hemostatic materials showed superior safety in a human level with liver tissue healing [26]. Subsequently, the series of tissue/material reactions promote granulations and re-organization, resulting in accelerated wound healing. Thus, CHI-C is beneficial for rapid healing, which might be responsible for its effective prevention of re-bleeding compared to the prevention efficacy of APC-involved healing.

**Reviewer #2:** *Interesting study with promising results...*

1. *Further details and clarifications about the CHI-C hemostatic agent preparation, characterization, properties. must be provided (substitution degree, sterilization, etc.). If all these details are provided in a previously published manuscript, it should be clearly indicated in the text*

**Answer**:

We apologize for the missing of further details and clarifications about the CHI-C hemostatic agent. As following the reviewer’s comments, we added the preparation, characterization and properties of CHI-C as follows:

[Figure 1]

[Before]

[After]

[Page 4, Lines 74-80]

[Before]

**Fig 1. Mussel adhesive proteins and mussel-inspired chitosan-catechol hemostatic agent.** (A) The coexistence of catechol (DOPA) and amine (lysine) groups in mussel adhesive proteins found in mussel threads is crucial for its underwater adhesion. (B) Mussel-inspired hemostatic agent (CHI-C) developed by chemically conjugating catechol groups to amine-rich chitosan. This material could be applied as a new method for endoscopic hemostasis.

[After]

**Fig 1**. **Mussel adhesive proteins and mussel-inspired chitosan-catechol hemostatic agent.** (A) The coexistence of catechol (DOPA) and amine (lysine) groups in mussel adhesive proteins found in mussel threads is crucial for its underwater adhesion. (B) Synthesis and chemical structure of CHI-C. (C) UV-vis spectrum of the CHI-C solution. (D) ^1^H-NMR spectrum of CHI-C dissolved in D_2_O. (E) Mussel-inspired hemostatic agent (CHI-C) developed by chemically conjugating catechol groups to amine-rich chitosan, and (F) an endoscopic photograph of the CHI-C hemostatic agent applied to gastric ulcer bleeding.

[Page 4, Lines 93-109]

[Added]

**Preparation of CHI-C hemostatic agent**

CHI-C was synthesized using standard EDC (1-Ethyl-3-(3-dimethylaminopropyl)-carbodiimide hydrochloride) chemistry as previously reported [26,27]. Briefly, 3 g of chitosan (100 mPa·s and 80% deacetylated, Heppe Medical Chitosan, Germany) was dissolved in 100 mL of HCl solution (pH 5). 3,4-Dihydroxyhydrocinammic acid (HCA, 2.37 g) and EDC (2.02 g) were dissolved in 25 mL of deionized and distilled water (DDW). The HCA/EDC solution was slowly added to the chitosan solution. During the coupling reaction, the pH value was slowly changed. Thus, it is important to monitor the pH value and to adjust it around 5.0. After 12 hours, the solution was dialysis (molecular weight cut-off: 12,000 to 14,000, SpectraPor, USA) against phosphate- buffered saline (pH 4) for 2 days and then the dialysis solution was changed to DDW for additional 4 hours. Then, the purified solution was freeze-dried. The degree of catechol substitution (DOC) was determined by UV-vis spectroscopy (Hewlett Packard 8453, Agilent) and Nuclear Magnetic Resonance (1H-NMR) spectroscopy (Bruker Avance, 400 MHz, D_2_O) as previously reported [17,26]. The CHI-C hemostatic material was prepared by a lyophilization method. CHI-C solution (1 wt%) was dissolved in DDW, and then transferred into polyethylene terephthalate molds. The CHI-C solutions in the molds were lyophilized for 3 days.

[Page 11, Lines 241-252]

[Added]

**Characteristics of CHI-C hemostatic agent**

We synthesized CHI-C using standard EDC chemistry with HCA (Fig 1B). The DOC value was determined using the absorbance of catechol at 280 nm by UV-vis spectroscopy (Fig 1C) in which HCA was used as a standard compound. The DOC in CHI-C was ~8.9 % of the total amine group in chitosan. ^1^H-NMR was also used to confirm the DOC value by comparing the ratio of the relative integral values for the catechol protons (3H at 6.67 ppm) to those of N-acetyl group protons (3H at 1.95 ppm) in chitosan backbone (80% deacetylated) (Fig 1D). The DOC in CHI-C calculated by ^1^H-NMR spectrum was ~10.3%. The error in DOC values might be originated from a little deviation of the deacetylation value of chitosan provided by the manufacturer. The CHI-C was dissolved in DDW at a concentration of 1 wt% and lyophilized in rectangular molds, which generated a porous CHI-C hemostatic agent for endoscopic application (Figs 1E and 1F).

2. *I believe that the follow-up endoscopy should have been blinded. Given that APC and CHI-C results are somewhat similar and that the study has other limitations specified by the authors, I would tone down the last sentence in the abstract by replacing outstanding by promising.*

**Answer**:

Thank you for your valuable comments. The follow-up endoscopy was performed in a non-blinded method. The main reason is that Sakita-Miwa classification (table 1) is highly objective scoring. Nonetheless, the authors tried to keep the scoring similar to a blinded situation during the entire experimental procedures by exchanging the results (i.e. cross-confirmation) between the two MDs (I.K. Yoo and G. Song) obtaining separate scores.

In addition, as for following the reviewer’s opinion about the last sentence in the abstract, we revised it as follows.

[Page 2, Lines 40-41]

[Before]

The new adhesive chitosan-catechol hemostatic agent shows outstanding results in gastrointestinal bleeding cases even for anticoagulant-treated pigs.

[After]

Considering the results, chitosan-catechol is an effective hemostatic material with reduced re-bleeding and improved healing.

3. *Please clarify if histological analysis was blinded or non-blinded.*

**Answer**:

The histological data were blindly examined by a histopathologist for the test materials. We added this information to the Material and Method section as follows:

[Page 10, Lines 221-222]

[Added]

The tissue sections were blindly examined by a histopathologist for the test materials.

4. *Specific comment about the Conclusions: The mention of clinical trials in the last sentence is premature in my opinion, more pre-clinical studies will have to be performed prior to initiate any clinical assessment and this sentence must be revised.*

**Answer**:

We are thankful for the reviewer’s comments. CHI-C hemostatic agent is already studied in human. Therefore, we added this information to the Conclusion section as follows:

[Page 18, Lines 398-404]

[Before]

In conclusion, CHI-C has hemostatic efficacy to stop Forrest Ib GI bleeding in a porcine model receiving anticoagulant therapy. This could be a new endoscopic treatment for GI bleedings. Future well-designed clinical trials with CHI-C after gastric EMR and endoscopic submucosal dissection (ESD) should be followed to demonstrate the benefits of its hemostatic effect.

[After]

In conclusion, CHI-C has hemostatic efficacy to stop Forrest Ib GI bleeding in a porcine model receiving anticoagulant therapy. This could be a new endoscopic treatment for GI bleedings. In previous exploratory clinical study, CHI-C hemostatic agent effectively staunched the oozing hemorrhage that cannot be controlled by primary hemostasis during hepatectomy in humans [26]. Future well-designed clinical trials with CHI-C after gastric EMR and endoscopic submucosal dissection (ESD) should be followed to demonstrate the benefits of its hemostatic effect.

*According to PLOS Data policy,*

*The data should be provided as part of the manuscript or its supporting information, or deposited to a public repository. For example, in addition to summary statistics, the data points behind means, medians and variance measures should be available. If there are restrictions on publicly sharing data—e.g. participant privacy or use of data from a third party— those must be specified.*

*Some data points are missing as its mostly summary statistics that are provided, it therefore seems that the authors didn't fully comply with this policy*

*Typos:*

*Sentence on line 171 is to be revised (was more severe or milder)*

*While revising your submission, please upload your figure files to the Preflight Analysis and Conversion Engine (PACE) digital diagnostic tool,* [*https://pacev2.apexcovantage.com/*](https://pacev2.apexcovantage.com/)*. PACE helps ensure that figures meet PLOS requirements. To use PACE, you must first register as a user. Registration is free. Then, login and navigate to the UPLOAD tab, where you will find detailed instructions on how to use the tool. If you encounter any issues or have any questions when using PACE, please email PLOS at figures@plos.org. Please note that Supporting Information files do not need this step.*

*In compliance with data protection regulations, you may request that we remove your personal registration details at any time. (Remove my information/details). Please contact the publication office if you have any questions.*
